# Neuroimmunomodulatory and Neuroprotective Effects of the Flavonoid Apigenin in *in vitro* Models of Neuroinflammation Associated With Alzheimer’s Disease

**DOI:** 10.3389/fnagi.2020.00119

**Published:** 2020-05-15

**Authors:** Naiara Silva Dourado, Cleide dos Santos Souza, Monique Marylin Alves de Almeida, Alessandra Bispo da Silva, Balbino Lino dos Santos, Victor Diogenes Amaral Silva, Adriano Martimbianco De Assis, Jussemara Souza da Silva, Diogo Onofre Souza, Maria de Fatima Dias Costa, Arthur Morgan Butt, Silvia Lima Costa

**Affiliations:** ^1^Laboratory of Neurochemistry and Cellular Biology, Institute of Health Sciences, Av. Reitor Miguel Calmon S/N, Federal University of Bahia (UFBA), Salvador, Brazil; ^2^Sheffield Institute of Translational Neuroscience (SITraN), The University of Sheffield, Sheffield, United Kingdom; ^3^College of Nursing, Federal University of Vale do São Francisco (UNIVASF), Petrolina, Brazil; ^4^INCT for Excitotoxicity and Neuroprotection (INCT-EN, BR), Porto Alegre, Brazil; ^5^Postgraduate in Health and Behavior, Catholic University of Pelotas (UCPEL), Pelotas, Brazil; ^6^Department of Biochemistry, Institute of Basic Health Sciences, Federal University of Rio Grande do Sul (UFRGS), Porto Alegre, Brazil; ^7^Instituto Nacional de Ciência e Tecnologia em Excitotoxicidade e Neuroproteção (INCT)—Translational Neuroscience (INCT-TN, BR), Porto Alegre, Brazil; ^8^School of Pharmacy and Biomedical Sciences, University of Portsmouth, Portsmouth, United Kingdom

**Keywords:** neuroinflammation, neuroprotection, anti-inflammatory, microglia, flavonoids

## Abstract

Neurodegenerative disorders (ND) are characterized by the progressive and irreversible loss of neurons. Alzheimer’s Disease (AD) is the most incident age-related ND, in which the presence of a chronic inflammatory compound seems to be related to its pathogenesis. Different stimuli in the central nervous system (CNS) can induce activation, proliferation, and changes in phenotype and glial function, which can be modulated by anti-inflammatory agents. Apigenin (4,5,7–trihydroxyflavone) is a flavonoid found in abundance in many fruits and vegetables, that has shown important effects upon controlling the inflammatory response. This study evaluated the neuroprotective and neuroimmunomodulatory potential of apigenin using *in vitro* models of neuroinflammation associated with AD. Co-cultures of neurons and glial cells were obtained from the cortex of newborn and embryonic Wistar rats. After 26 days *in vitro*, cultures were exposed to lipopolysaccharide (LPS; 1 μg/ml), or IL-1β (10 ng/ml) for 24 h, or to Aβ oligomers (500 nM) for 4 h, and then treated with apigenin (1 μM) for further 24 h. It was observed that the treatment with apigenin preserved neurons and astrocytes integrity, determined by Rosenfeld’s staining and immunocytochemistry for β-tubulin III and GFAP, respectively. Moreover, it was observed by Fluoro-Jade-B and caspase-3 immunostaining that apigenin was not neurotoxic and has a neuroprotective effect against inflammatory damage. Additionally, apigenin reduced microglial activation, characterized by inhibition of proliferation (BrdU+ cells) and modulation of microglia morphology (Iba-1 + cells), and decreased the expression of the M1 inflammatory marker CD68. Moreover, as determined by RT-qPCR, inflammatory stimuli induced by IL-1β increased the mRNA expression of IL-6, IL-1β, and CCL5, and decreased the mRNA expression of IL-10. Contrary, after treatment with apigenin in inflammatory stimuli (IL-1β or LPS) there was a modulation of the mRNA expression of inflammatory cytokines, and reduced expression of OX42, IL-6 and gp130. Moreover, apigenin alone and after an inflammatory stimulus with IL-1β also induced the increase in the expression of brain-derived neurotrophic factor (BDNF), an effect that may be associated with anti-inflammatory and neuroprotective effects. Together these data demonstrate that apigenin presents neuroprotective and anti-inflammatory effects *in vitro* and might represent an important neuroimmunomodulatory agent for the treatment of neurodegenerative conditions.

## Introduction

Neurodegenerative disorders (ND) age-related represent a serious public health problem in which incidence has increased due to augmented population aging. These disorders are associated with the progressive loss of neurons (Procaccini et al., 2016) and studies suggest that exacerbated inflammatory response could be the major cause behind neurodegeneration (Doty et al., 2015).

Among the cells that comprise the central nervous system (CNS), astrocytes and microglia have been proved for playing a critical role in physiology and disease (Allen and Barres, 2009), because these glial cells are capable to respond actively against toxins, infections and injuries to the CNS (Burda and Sofroniew, 2014; Kinney et al., 2018). Glial activation is a protective mechanism that regulates tissue repair in the early stage of neurodegeneration (Streit, 2005). However, excessive and prolonged activation contributes to a chronic neuroinflammatory response (Kraft and Harry, 2011), that might be involved in the onset and progression of most ND, such as Amyotrophic lateral sclerosis, Parkinson’s disease and Alzheimer’s disease (Heneka et al., 2015; Ghadery et al., 2017; Shi and Holtzman, 2018; Jara et al., 2019; McCauley and Baloh, 2019). According to van Horssen et al. (2019), microglial and astrocytic activation leads to the production of inflammatory mediators such as cytokines, chemokines, reactive oxygen and nitrogen species, which eventually contribute to neuronal death.

Beyond the diseases that imply greater socioeconomic impact, Alzheimer’s disease (AD) is the most frequent neurodegenerative disorder in the world characterized by the accumulation of β-amyloid (Aβ) protein in the brain parenchyma, the formation of neurofibrillary tangles, glial activation and production of inflammatory mediators such as NO, interleukin 1β (IL-1β), interleukin 6 (IL-6) and tumor necrosis factor-α (TNF-α; Zotova et al., 2013; Alasmari et al., 2018). Despite new therapeutic approaches carried out over the past few years, treatments for AD and other ND are still limited and do not stop the progression, but mainly control symptoms. Thus, targeting neuroinflammation represents one of the most promising disease-modifying treatments against ND.

Recently recognized for their powerful effect upon controlling inflammation, flavonoids have been largely studied as an alternative to treat inflammatory conditions. These compounds are derived from plant secondary metabolism and are widely distributed in the plant kingdom (Agati et al., 2011). They act by interfering with several intracellular processes, such as increasing the activation of antioxidant enzymes (Magalingam et al., 2013), additionally to the suppression of lipid peroxidation (Schinella et al., 2010) and inhibition of pro-inflammatory and proapoptotic mediators (Zhang et al., 2011; Raza et al., 2013). Studies suggest the correlation of dietary flavonoid consumption with the reduction of dementia levels (Beking and Vieira, 2010; Shahidi and Ambigaipalan, 2015; Terahara, 2015).

Apigenin (4,5,7–trihydroxyflavone) is a flavonoid belonging to the class of flavones, found in abundance in fruits and teas (McKay and Blumberg, 2006; Gazola et al., 2015). Studies have shown several biological activities associated with apigenin treatment, such as antioxidant (Han et al., 2012), anti-inflammatory (Lee et al., 2015), neurogenic (Souza et al., 2015), neuroprotective (Balez et al., 2016), and antitumor (Coelho et al., 2019). Importantly, apigenin can cross the blood-brain barrier (Popović et al., 2014), which is of great significance to treat CNS disorders. However, despite studies have shown its diverse biological activities in different models, the mechanisms by which apigenin promotes neuroprotection remain elusive.

In this light, in this study, we used a well-established co-culture model of neurons and glial cells (Silva et al., 2013) to investigate the anti-inflammatory and neuroprotective activity of the flavonoid apigenin, in three different models of neuroinflammation induced by lipopolysaccharide (LPS; classic neuroinflammation), IL-1β or Aβ oligomers. Overall, it was demonstrated that apigenin is not neurotoxic in the tested concentration and has neuroprotective potential, evidenced by the reduction of caspase 3 activation and the increase in neuronal viability, and this effect is thought to be mainly through the control of microglia and astrocyte inflammatory response.

## Materials and Methods

### Neuron/Glial Cells Co-cultures

**Figure 1:**
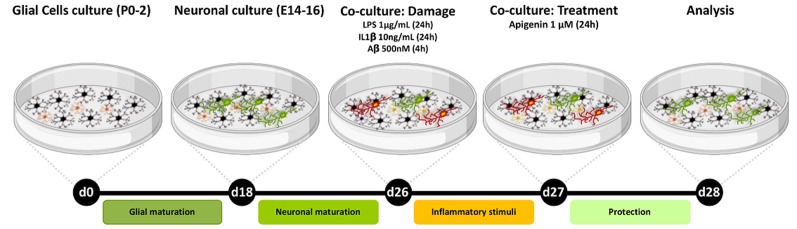
Experimental design. Neurons/glia co-cultures were obtained from the cortex of Wistar rats. After 26 days of cultivation, the cultures were treated with either Aβ oligomers (500 nM) for 4 h or IL-1β (10 ng/ml) or LPS (1 μg/ml) for 24 h and then treated with apigenin (1 μM) and analyzed after 24 h treatments.

Glial cells and neurons were obtained from the brain hemispheres of Wistar rats as described previously (Dos Santos Souza et al., 2018). The animals were provided by the Animal Facilities of the Department of Physiology of the Institute of Health Sciences of the Federal University of Bahia (Salvador, Brazil). All experiments were performed following the local Ethical Committee for Animal Experimentation of the Health Sciences Institute (CEUA, Protocol n°027/2012). For the co-cultures, glial cells were obtained from the cortex of neonate animals, aged 1 to 2 days, and neurons were obtained from embryos with 14–16 day-old embryos (Figure 1). Cerebral hemispheres were removed aseptically. Meninges and blood vessels were removed from each cortex, and then the material was mechanically dissociated and filtered into a sterile 75 mm diameter Nitex membrane (R&D^®^). Dissociated cells were then cultured in DMEM HAM F12 medium (Gibco^®^), supplemented with amino acids (2 mM L-glutamine and 0.011 g/l pyruvate, Merck), 10% fetal bovine serum (FBS, Gibco^®^), 3.6 g/l Hepes (Merck), 33 mM glucose (Cultilab, SP, Brazil), antibiotics (100 IU/ml penicillin G and 100 μg/ml streptomycin, Gibco^®^), and cultured in 100-mm Ø plates (TPP) in a humidified atmosphere with 5% CO_2_ at 37°C. The culture medium was changed every 48 h and cells were cultured for 15 days. Cells were then washed three times with PBS, detached with trypsin solution at 37°C (Trypsin/EDTA, Merck), plated at a density of 1 × 10^5^ cell/cm^2^ as described previously (Dos Santos Souza et al., 2018) and maintained in culture for 72 h. After incubation, neurons obtained from cerebral hemispheres of 14- to 16-day-old Wistar rat embryos, using the same method described above for glial isolation, were suspended in supplemented DMEM/HAM F12 (Gibco^®^) and seeded at half the number of glial cells (5 × 10^4^ cells/cm^2^) onto the glial monolayer. The co-cultures were incubated in a humidified atmosphere with 5% CO_2_ at 37°C for 8 days *in vitro* and the medium changed every 48 h.

### Drugs and Treatments

Flavonoid apigenin (4′,5,7-trihydroxyflavone) adopted in this work was purchased commercially (Sigma–Aldrich, St. Louis, MO, USA 97% purity A3145). It was dissolved in dimethyl sulfoxide (DMSO, Sigma–Adrich, St. Louis, MO, USA) to a stock concentration of 100 mM and kept protected from light at a temperature of −20°C. Final dilution was obtained at the time of treatment by diluting the concentrated solution directly into the culture medium. Cells were exposed to flavonoids at a final concentration of 1 μM. Control cultures were treated with DMSO in a volume equivalent to apigenin concentration (0.01%). Experimental analyses were performed 24 h after the treatment. To induce inflammatory damage, co-cultured cells were exposed for 24 h to LPS (1 μg/ml, Sigma Chemical Company L2880) or Interleukin 1 beta (IL-1β, 10 ng/ml; R&D Systems 501-RL-010), or for 4 h to Aβ oligomers (500 nM, American Peptide). The experimental design is illustrated in Figure 1. Final dilution of LPS and IL-1β was obtained at the time of treatment by diluting the stock solution directly into the culture medium. The concentration and exposure time adopted followed established protocols (Radesäter et al., 2003; Moraes et al., 2015). Solubilization of the β-amyloid peptide from synthetic Aβ1–42 peptide (American Peptide) was performed according to protocol already established (De Felice et al., 2008; Lourenco et al., 2013), and was diluted in culture medium to obtain a 500 nM solution from a stock solution (100 μM). The concentration and exposure time adopted followed established protocols described in the literature (Lourenco et al., 2013). In brief, Aβ1–42 peptide was solubilized at 1 mM in ice-cold 1,1,1,3,3,3 hexafluoro-2-propanol (HFIP; Merck) and the resulting clear colorless solution was incubated at room temperature for 60 min. The solution was then placed on ice for 10 min and aliquoted (25 μl of HFIP solution to obtain 0.133 mg Aβ). Microtubes were left open in the laminar flow hood for 12 h for evaporation of HFIP. The complete elimination of HFIP was done by SpeedVac^®^ centrifugation for 10 min. Aliquots containing Aβ films were stored at −20°C for later use. Aβ oligomer preparations were made from Aβ films resuspended in 2% dimethylsulfoxide (DMSO; Sigma-Adrich, St. Louis, MO, USA) to obtain a solution at 5 mM. This solution was then diluted in 100 μM sterile PBS and incubated at 4°C for 24 h. After incubation, the preparation was centrifuged at 14,000 *g* for 10 min at 4°C to remove insoluble Aβ aggregates (fibrils). The centrifugation supernatant containing the oligomers was kept at 4°C until use. To determine the concentration of oligomers in the preparations, the BCA Kit (BIO-RAD) was used.

### Fluoro-Jade B Staining

The neuroprotective potential of apigenin was assessed with the Fluoro-Jade B assay (FJB, Millipore, AG310). This staining was used to evaluate neuronal death. Cells were cultured in 96-well black bottom plates (Corning Incorporated, 3603) and treated as described. After treatments the co-culture, supernatants were removed and the cells were fixed with ethanol at 4°C for 10 min, washed three times with PBS, and permeabilized with 0.3% Triton X-100 in PBS (Merck) for 10 min. After this time, the cultures were washed three times with distilled water and incubated with 0.001% FJB solution for 30 min at room temperature (RT), under slow agitation and protected from the light. After incubation, the cells were washed three times with PBS and incubated for 5 min at RT in the dark with 5 μg/ml 4,6-diamidino-2-phenylindole (DAPI) for nuclear staining, and then washed three times with PBS. Analyses were performed on a spectrophotometer (Varioskan™ Flash Multimode Reader, Thermo Plate), and the fluorescence intensity of each sample was measured at 480 nm for FJB and 350 nm for DAPI. The value of absorbance of FJB of each well was normalized to the DAPI absorbance in the same well. Three independent experiments were performed. Thereafter, cells were analyzed using a fluorescence microscope (Leica, DFC7000). Quantification was analyzed with ImageJ 1.33u.

### Rosenfeld’s Staining

**Figure 2:**
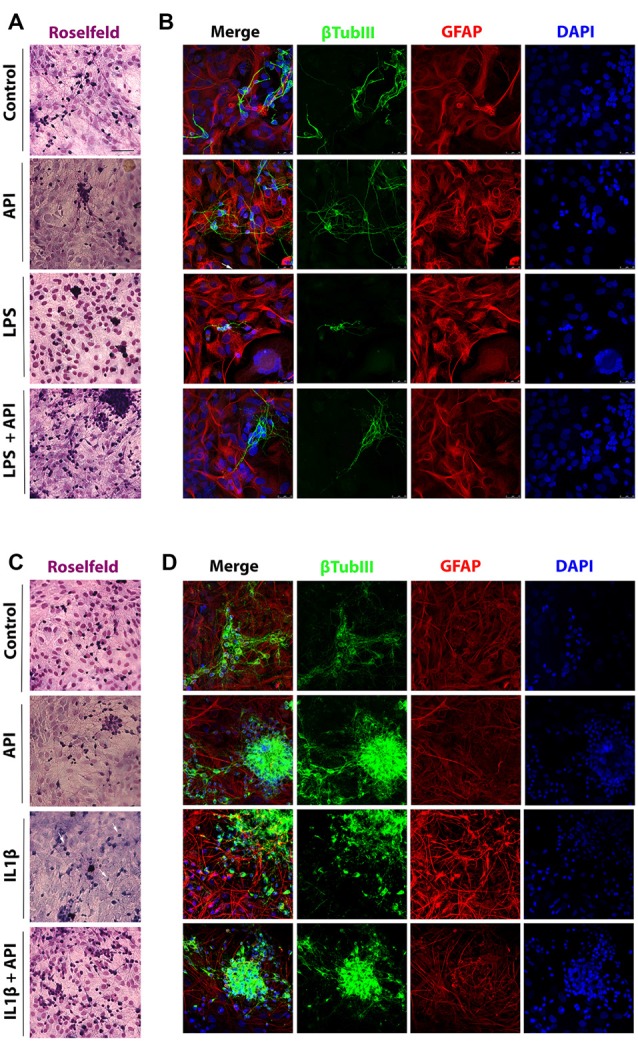
Apigenin preserves neuronal and astrocyte morphology. Effects of apigenin (API, 1 μM) treatment on the integrity of neurons and astrocyte reactivity after lipopolysaccharide (LPS; (**A,B**, 1 μg/ml) and IL-1β (**C,D**, IL-1β 10 ng/ml) inflammatory stimuli. Morphological changes were primarily assessed by analyzing the Rosenfeld’s staining **(A,C)**. The patterns of expression of the cytoskeletal protein β-tubulin III (β-tubIII, green) specific of neurons, and the cytoskeletal protein GFAP (red) marker of astrocyte morphology and reactivity were determined by immunocytochemistry and confocal analysis after 24 h treatments; control cultures were treated with dimethyl sulfoxide (DMSO; 0.01%) and nuclear chromatin was stained with 4,6-diamidino-2-phenylindole (DAPI; blue). Obj. 40×, scale bar: 50 μm; white arrows indicate neurons without neurites.

Glial and neuronal morphological changes in response to inflammatory stimuli and treatment with apigenin were primarily assessed by Rosenfeld’s staining. After exposure of the cells to the treatments, the culture medium was discarded and the cells were washed three times with phosphate-buffered saline (PBS, Sigma) and then fixed in 4% paraformaldehyde for 20 min at room temperature (RT), after that, cultures were washed three times with PBS. Fixed cells were stained by the protocol established by Rosenfeld (Rosenfeld, 1947). Rosenfeld’s reagent was added and incubated for 20 min at room temperature. Three independent experiments were performed. Thereafter, the plates were rinsed with water, air-dried, analyzed in an optic phase microscope (Nikon; Figures 2A,C).

### Immunocytochemistry

Glial and neuronal response to inflammatory stimuli and treatment with apigenin was assessed by immunocytochemistry using the following primary antibodies: anti-β-Tubulin III (mouse, 1:500; BioLegend, 801202), anti-GFAP (rabbit, 1:300; DAKO, Z0334), anti-Iba-1 (ionized calcium-binding adaptor molecule 1; rabbit, 1:200; Wako, 019-19741), anti-CD68 (rat, 1:100; Abcam, ab53444), anti-active caspase-3 (rabbit, 1:300; Chemicon, ab3623), anti-CD11b/c (OX42; mouse, Abcam, ab1211), anti-IL-6 (rabbit, 1:500; Abcam, ab6672) and anti-gp130 (rabbit, 1:500; Abcam, ab6672). After exposure of the cells to the treatments, the culture medium was discarded and the cells were washed three times with phosphate-buffered saline (PBS, Sigma) and then fixed in 4% paraformaldehyde for 20 min at room temperature (RT). After that cultures were washed three times with PBS and permeabilized with Triton X-100 (0.3%) in PBS for 15 min and then washed again with PBS three times for 5 min. After this time, non-specific binding sites were blocked by incubation with PBS containing 5% bovine serum albumin (BSA; Sigma–Adrich, St. Louis, MO, USA) for 1 h. After blocking, samples were incubated with primary antibodies diluted in PBS containing 1% of BSA in a humid chamber at 4°C for 12 h. Subsequently, cells were washed three times with PBS and incubated with secondary antibodies diluted in PBS containing 1% of BSA and kept under slow agitation for 1 h at RT, protected from the light. The cells were washed with PBS three times and incubated with 5.0 μg/ml 4,6-diamidino-2-phenylindole (DAPI, Molecular Probes, Eugene, OR, USA) for nuclear staining for. The cell coverslips were then washed three times in PBS and mounted on slides containing 80% glycerol N-propyl gallate mounting medium (Sigma–Adrich, St. Louis, MO, USA). Staining was visualized on a fluorescence microscope (Leica, DFC7000) and (Leica, SP8 confocal). Images were captured with a 20× or 40× objective. Three independent experiments were performed. Quantification was analyzed with ImageJ 1.33u. The following secondary antibodies were used at the indicated dilutions: Alexa Fluor 488-conjugated goat anti-mouse IgG (1:500; Molecular Probes, A11001), Alexa Fluor 594-conjugated goat anti-rabbit IgG (1:500; Molecular Probes, A11012), Alexa Fluor 488 goat anti-rabbit IgG [H&L] (1:500; Molecular Probes A11008), Alexa Fluor 555-conjugated goat anti-rat IgG (1:500; Molecular Probes, A21434) and Alexa Fluor 594-conjugated goat anti-rabbit IgG (1:500, Molecular Probes A11012). The quantification was performed by analyzing the total number of positive cells (per marker), divided by the total number of nuclei (DAPI positive) × 100.

### Bromodeoxyuridine Cell Proliferation Assay

To evaluated proliferation was using the Bromodeoxyuridine (BrdU) cell proliferation assay (Sigma–Aldrich, Inc., St. Louis, MO, USA). BrdU (10 μM) was added to the wells since each treatment had started. Cells were incubated for 2 h in a humidified atmosphere of 95% air and 5% CO_2_ at 37°C. Cells were fixed and DNA was denatured by treatment with denaturing solution (2 N HCl) for 20 min at room temperature. Anti-BrdU monoclonal antibody (mouse, 1:200, Sigma–Adrich, St. Louis, MO, USA B8434) diluted in PBS was pipetted into the wells and allowed to incubate for 1 h. Unbound antibody was washed away and cells were incubated with Alexa Fluor 546-conjugated goat anti-mouse IgG (1:500; Molecular Probes, A11003), diluted in PBS-T for 1 h under slow agitation at room temperature. After incubation, the cell nuclei were stained with DAPI (5 μg/ml) for 10 min at room temperature. All reagents were used following the manufacturer’s instructions. Experiments were performed in triplicate. Thereafter, cells were analyzed using a fluorescence microscope (Leica, DFC7000) Quantification was analyzed with ImageJ 1.33u software (Wayne Rasband, National Institutes of Health, Bethesda, MD, USA).

### Quantitative RT-PCR

To evaluate gene expression for proteins of interest, after the treatment period, the culture medium was removed and then total RNA was extracted with Trizol^®^ reagent (Invitrogen, Life Technologies, 15596026). Extraction was performed according to the manufacturer’s specifications. Total RNA purity and concentration were determined by spectrophotometric analysis using KASVI Nano Spectrum (cat# K23-0002). DNA contaminants were removed by treating the RNA samples with DNase using the Ambion DNA-free kit (cat# AM1906, Life Technologies™). For cDNA synthesis, SuperScript^®^ VILO™MasterMix (cat# MAN0004286, Invitrogen™, Life Technologies) was used in a 20-μl reaction with a concentration of 2.5 μg of total RNA, following the manufacturer’s instructions. Quantitative real-time PCR (qPCR) was performed using Taqman^®^ Gene Expression Assays (Applied Biosystems, Foster City, CA, USA) containing two primers to amplify the sequence of interest, a specific Taqman^®^ MGB probe and TaqMan Universal Master Mix II with UNG (cat# 4440038 Invitrogen, Life Technologies™). The assays corresponding to the genes quantified in this study were IL-1β (Rn00580432_m1), IL-6 (Rn01410330_m1), CCL5 (Rn00579590_m1), IL-10 (Rn00563409_m1), brain-derived neurotrophic factor (BDNF; Rn02531962_s1), and NTF4 (Rn00566076_s1). Real-time PCR was performed using the Quant Studio 7 Flex™Real Time PCR System (Applied Biosystems, CA, USA). The thermocycling conditions were performed according to the manufacturer’s specifications. The actin beta (Actb; Rn00667869_m1) and hypoxanthine phosphoribosyltransferase 1 (HPRT1; Rn01527840_m1) targets were used as reference genes (endogenous controls) for normalization of gene expression data. Data were analyzed using the 2^−ΔΔCt^ method. The results represent the average of three independent experiments.

### Statistical Analyses

The results were analyzed by the GraphPad Prism 5, statistical program (CA, USA). It was analyzed whether the values came from a Gaussian distribution. To determine the statistical difference between the groups, we performed one-way analysis of variance (ANOVA) followed by Student-Newman–Keuls post-test for normal samples. For non-normal samples, an analysis was performed using Kruskal–Wallis followed by Dunn’s post-test. Confidence intervals were defined at a 95% confidence level (*p* < 0.05 was considered to be statistically significant). Fold change was calculated by dividing the average (mean) value of the experimental group by that of the control group. In all figures, error bars represent SEM of at least three independent experiments.

## Results

### Apigenin Preserves Neuron and Astrocyte Morphology After LPS and IL-1β-Induced Inflammatory Stimuli

LPS a component of the gram-negative bacteria cell membrane, is known to be a potent inducer of the inflammatory response *via* toll-like receptor 4 (Park and Lee, 2013; Xu et al., 2017) and according to Jin et al. (2017), the IL-1β is a proinflammatory cytokine that plays a key role in the initiation and development of a complex cellular inflammatory cascade. Excessive IL-1β production in the CNS, mainly by glial cells, is associated with neuroinflammation found in neurodegenerative processes. Here, we investigate the effects of flavonoid apigenin against classical neuroinflammation produced by LPS and against inflammatory damage produced by cytokine IL-1β in glia/neurons co-cultures in terms of preservation of neuronal integrity, modulation of glial (astrocyte and microglia) activation and inflammatory signaling (Figure 2). Glial and neuronal Morphological changes in response to inflammatory stimuli and treatment with apigenin were primarily assessed by Rosenfeld’s staining a differential stain used to differentiate nuclear and cytoplasmic morphology (Figures 2A,C). We observed that in control conditions (0.01% DMSO), the glial cells represented by light pink cytoplasm and dark pink big nucleus, the cells look diffused with a polygonal shape. On the other hand, neurons are represented by a dark purple cytoplasm with neurites represented by long and fine purple processes. We do not observe morphological changes in cells treated with apigenin when compared with control cells. However, we observed a reduction in the neurites in the neurons after the treatment with LPS (Figure 2A). On the other hand, apigenin was able to rescue the neuronal integrity after LPS treatment. Similar results were observed after the treatment with IL-1β, where we can observe a reduction in the neurites induced by IL-1β and a rescue for apigenin (Figure 2C).

To evaluate the effect of apigenin treatment on neurons integrity after 24 h treatment with LPS or with IL-1β, immunostaining was performed for β-tubulin III (β-tubIII) a standard neuronal marker in association with GFAP, a classical marker of astrocyte reactivity. We observed that apigenin treatment (1 μM; Figures 2B,D) preserved the neurites network, maintained neuronal cell body integrity and typical astrocyte polygonal morphology, similar to the control condition (0.01% DMSO). After induction of inflammatory stimulus with LPS (1 μg/ml), the neurites network was not preserved, and only perinuclear staining was observed, associated with cell bodies forming irregular clustering (Figure 2B). Also, astrocytes showed reactive morphology, characterized by the presence of longer processes with higher GFAP expression (Figure 2B). However, in cultures treated with apigenin after LPS damage astrocyte maintained the non-reactive phenotype (similar to that observed under control conditions and after apigenin treatment) and the neurons integrity was preserved, as well as their networks of interconnections, compared to cultures treated with LPS alone.

The effects of apigenin on the integrity of neurons and astrocytes were confirmed in co-cultures submitted to IL-1β-induced neuroinflammation (Figure 2D). After induction of IL-1β stimulus (10 ng/ml), few cells labeled for βtubIII were observed when compared to the control cultures. Also, it was verified in astrocytes the emission of cellular processes with high immunoreactivity to GFAP, characteristic of reactive astrocytes. However, apigenin treatment after IL-1β damage was able to maintain the entire neurite network, increase the intensity of the βtubIII labeling with neuron cluster formation, and preserve the astrocyte morphology similar to that observed in the control conditions (Figure 2D).

### Apigenin Protects Neurons Against LPS and IL-1β-Induced Inflammatory Stimuli

**Figure 3:**
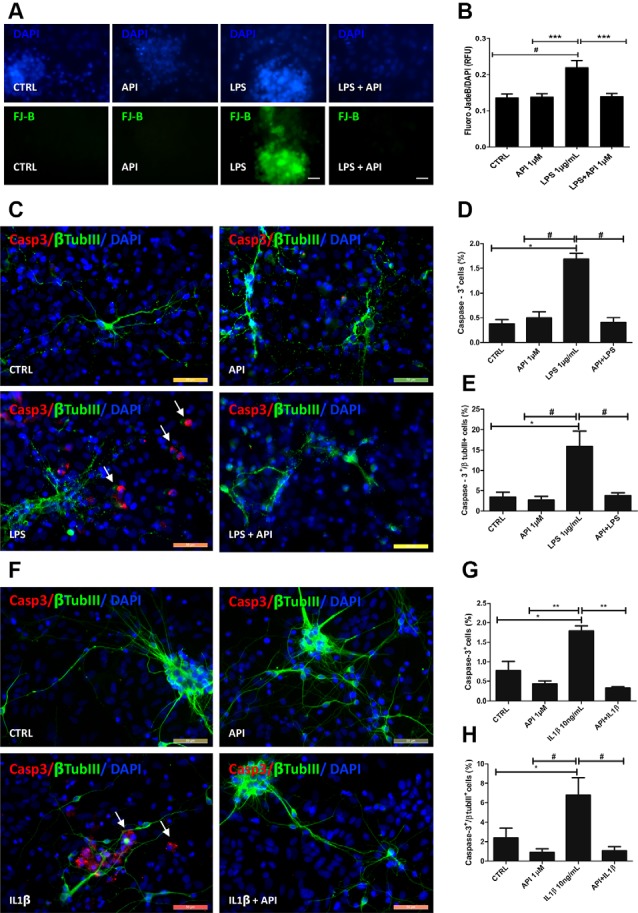
Apigenin protects neurons against neuroinflammation induced by LPS and IL-1β. Effects of apigenin (API, 1 μM) treatment on the integrity of neurons and induction of apoptosis after lipopolysaccharide (LPS) (**A–E**, 1 μg/ml) or IL-1β (**F–H**, 10 ng/ml) inflammatory stimuli. **(A)** Evaluation of neuronal degeneration by FJB assay in cultures after LPS (1 μg/ml) inflammatory stimuli. **(B)** Graph Barr showing neuronal degeneration by Fluoro-Jade B (FJB) assay. **(C,F)** The patterns of expression of the cytoskeletal protein β-tubulin III (β-tubIII, green) specific of neurons, and of the activated caspase-3 (CASP, red), a marker of cells in apoptosis, were determined by immunocytochemistry after 24 h treatments. Control cultures were treated with DMSO (0.01%) and nuclear chromatin was stained with DAPI (blue). **(D,G,E,H)** Graphs represent the total population of caspase-3 positive cells, and the population of cells doubly labeled βTubIII and caspase-3; *statistical significance concerning control (DMSO 0.01%), *p*-value < 0.01; ^#^statistical significance in relation to the group treated with LPS, *p*-value < 0.01; ** and ^#^statistical significance in relation to the group treated with IL-1β *p*-value < 0.001 and *p*-value < 0.01; Statistical test: analysis of variance (ANOVA) followed by Student Newman–Keuls test; results expressed as mean ± standard deviation of FJB fluorescence intensity; ^#^represents statistical difference compared to control DMSO 0.01%) with *p*-value < 0.001 ***represents statistical difference compared to the group treated with LPS with *p*-value < 0.001. Values are expressed as the mean ± SEM (*n* = 3) and were tested for significance by ANOVA followed by the Newman–Keuls test. Obj. 40×, scale bar: 50 μm; arrows indicate cells doubly labeled βTubIII and caspase-3.

To better characterize the neuroprotective potential of apigenin against neuroinflammation, glia/neurons were exposed to LPS (1 μg/ml) or IL-1β (10 ng/ml) for 24 h and then treated with apigenin (1 μM) for additional 24 h, and then labeled with Fluor Jade-B (FJB) that marks neurons in degeneration (Figures 3A,B). Apigenin treatment did not change neuronal viability when compared to control cultures (0.01% DMSO). On the other hand, induction of damage by LPS led to neuronal degeneration, with an increase of 0.61 ± 0.14 in the relative fluorescence intensity (IRF) compared to control. It was observed that after LPS damage, apigenin treatment reduced IRF to baseline levels observed in the control condition, also demonstrating its neuroprotective potential (Figures 3A,B).

To verify the neuroprotective potential of apigenin against neuroinflammation, immunocytochemistry evaluation of the presence of active caspase-3 a classical apoptosis marker in association with β-tubulin III (βtubIII) was performed (Figures 3C–H). The induction of damage by LPS promoted caspase-3 activation in 1.6 ± 0.1% of total cells, and 15.8 ± 3.7% of βtubIII positive neurons (Figures 3C–E). This increase was significant compared to the control [0.3 ± 0.08% and 3.4 ± 1.1%, respectively (Figures 3C–E)]. On the other hand, after LPS damage, apigenin treatment significantly reduced the proportion of caspase-3 positive cells (0.4 ± 0.09%) and caspase-3/βtubIII positive neurons (3.7 ± 0.7%) when compared to cultures treated with LPS alone, and no difference was observed between this group and the control group. IL-1β treatment promoted caspase-3 activation in 1.7 ± 0.1% of total cells and 7.6 ± 1.7% of βtubIII positive neurons compared to control cultures (0.7 ± 0.2% and 2.3 ± 1.0%, respectively; Figures 3F–H). However, apigenin treatment after IL-1β damage reduced the percentage of caspase-3 positive cells (0.3 ± 0.03%) and double-labeled neurons for caspase-3 and βtubIII (1.0 ± 0, 4%) when compared to cultures treated with IL-1β alone. Moreover, treatment with 1 μM apigenin did not induce caspase-3 activation compared to control (DMSO 0.01%). These data suggest that apigenin does not induce cell death in glia/neurons co-cultures and disrupts the progression of apoptosis in a neuroinflammation model. Together these findings demonstrate the neuroprotective and glial modulatory potential of the flavonoid in conditions of inflammatory stimuli and neuronal damage.

### Apigenin Modulates Microglial Activation Profile After Inflammatory Stimuli

**Figure 4:**
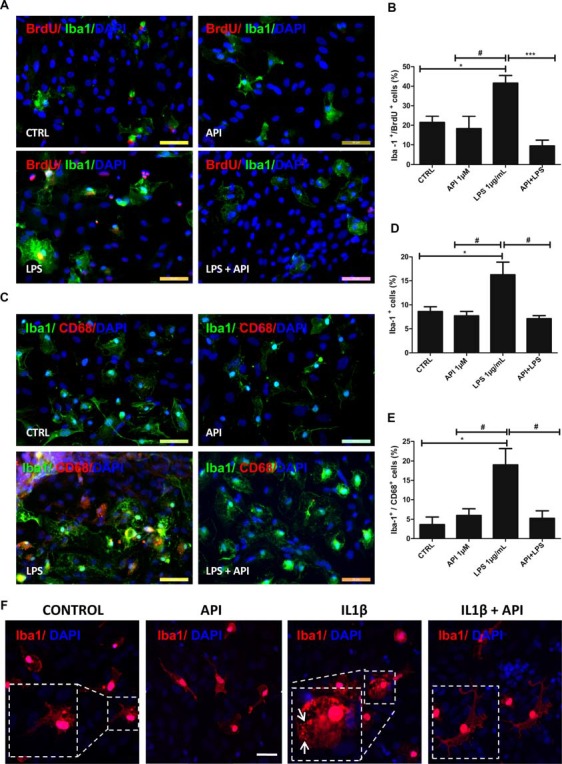
Apigenin modulates microglial activation profile. Effects of apigenin (API, 1 μM) treatment on the activation of microglia after LPS (**A–E**, 1 μg/ml), or IL-1β (**F**, 10 ng/ml) inflammatory stimuli in glia/neurons co-cultures. Proliferation and changes in morphology, both features of microglial activation were analyzed. **(A,B)** Immunocytochemistry for ionized calcium-binding adapter molecule 1 (Iba-1, green), specific of microglia, associated with bromodeoxyuridine (BrdU; red), that marker cells in proliferation was performed in cultures submitted to inflammatory stimuli with LPS. Graphs **(B,D)** represents cell population labeled for both Iba-1 and BrdU; data a represented as mean of percentage ± standard deviation of immunofluorescence labeling of Iba-1 and BrdU cells **(C)** Immunocytochemistry for Iba-1 (green), associated with CD68 (red), marker of activated microglia/macrophages in a proinflammatory profile was performed after 24 h treatments. **(D,E)** The graphs represent the total population of Iba-1 positive cells (microglia) and the population of double-labeled Iba-1 +/CD68 + microglia. **(F)** Immunocytochemistry for the cytoskeletal protein Iba-1 (red), was also performed in cultures submitted to inflammatory stimuli with IL-1β; control cultures were treated with DMSO (0.01%) and nuclear chromatin was stained with DAPI (blue); Obj. 40×, scale bar: 50 μm.; *represents statistical significance in relation to the control (DMSO 0.01%) with *p*-value < 0.01; ^#^represents statistically significant difference compared to the group treated with LPS, with *p*-value < 0.01 and ***represents statistical significance in relation to the group treated with LPS, with *p*-value < 0.001. Values are expressed as the mean ± SEM (*n* = 3) and were tested for significance by ANOVA followed by the Newman–Keuls test; arrows indicate Iba-1 positive cells showed ameboid morphology.

To evaluate if the neuroprotective effect of apigenin after different inflammatory stimuli is associated with microglial activation, first immunostaining was performed for Iba-1, a classic structural marker of microglia in co-cultures to characterize changes on morphology, and in association with BrdU immunostaining, to characterize microglia in proliferation. After induction of damage by LPS (1 μg/ml), the percentage of Iba-1 and BrdU positive cells increased (41.5 ± 4%) compared to the control (21.4 ± 3.2%), which characterizes microglial proliferation (Figures 4A,B). However, apigenin treatment after LPS damage significantly reduced the percentage of positive Iba-1 and BrdU cells (9.4 ± 2.8%) when compared to co-cultures treated with LPS alone and did not show statistical significance in the percentage of these cells when compared to control. Moreover, apigenin treatment (1 μM) had no significant effect on the proportion of double-labeled cells for Iba-1 and BrdU compared to control co-cultures.

Modulation of the microglial profile was also evaluated based on the morphological changes visualized by the Iba-1 protein immunostaining. We verified that under control conditions treated with 0.01% DMSO, the microglia presented branched morphology. Exposure to LPS (1 μg/ml; Figure 4A), was able to induce phenotypic changes. It was observed that in these conditions, Iba-1 positive cells showed ameboid morphology. However, microglia morphology in co-cultures treated with apigenin after inflammatory stimuli presented a morphological pattern similar to that observed in the control conditions, showing branched morphology.

Moreover, to evaluate the effects of apigenin on the modulation of the microglial profile after inflammatory stimuli with LPS (1 μg/ml), double immunostaining was performed for Iba-1 protein and CD68, a specific M1 profile marker in glia/neurons co-cultures (Figure 4C). After exposure to LPS, an increase in the proportion of Iba-1 positive cells (16.2 ± 2.6%; Figure 4D) and CD68 positive microglia (19 ± 4.1%) was observed compared to the control cultures (8.6 ± 0.9% and 3.6 ± 1.9%, respectively; Figure 4E). On the other hand, apigenin (1 μM) treatment after LPS damage reduced significantly the percentage of Iba-1 positive cells (7.1 ± 0.6%) and CD68 positive cells (5.2 ± 1.9%) when compared to cultures treated with LPS only (Figures 4C,E). No significant effect on the proportion of microglia and expression of the M1 marker was observed after apigenin treatment.

To confirm if the neuroprotective effect of apigenin after different inflammatory stimuli is associated with microglial activation, we stimulated the cells with IL-1β and then with apigenin. Similarly to LPS the treatment with IL-1β (10 ng/ml), was able to induce phenotypic changes in microglial cells (Figure 4F) evidenced by the ameboid morphology of Iba-1 positive cells. However, microglia morphology in co-cultures treated with apigenin after inflammatory stimuli induced by IL-1β (10 ng/ml), presented morphological pattern similar to that observed in the control conditions, showing branched morphology (Figure 4F). These results demonstrate that apigenin reduced microglial proliferation/activation after the inflammatory stimulus and reaffirm its anti-inflammatory effect.

### Apigenin Regulates the Expression of Inflammatory Mediators in Neurons/Glial Cells Co-cultures Submitted to IL-1β or LPS Stimulus

**Figure 5:**
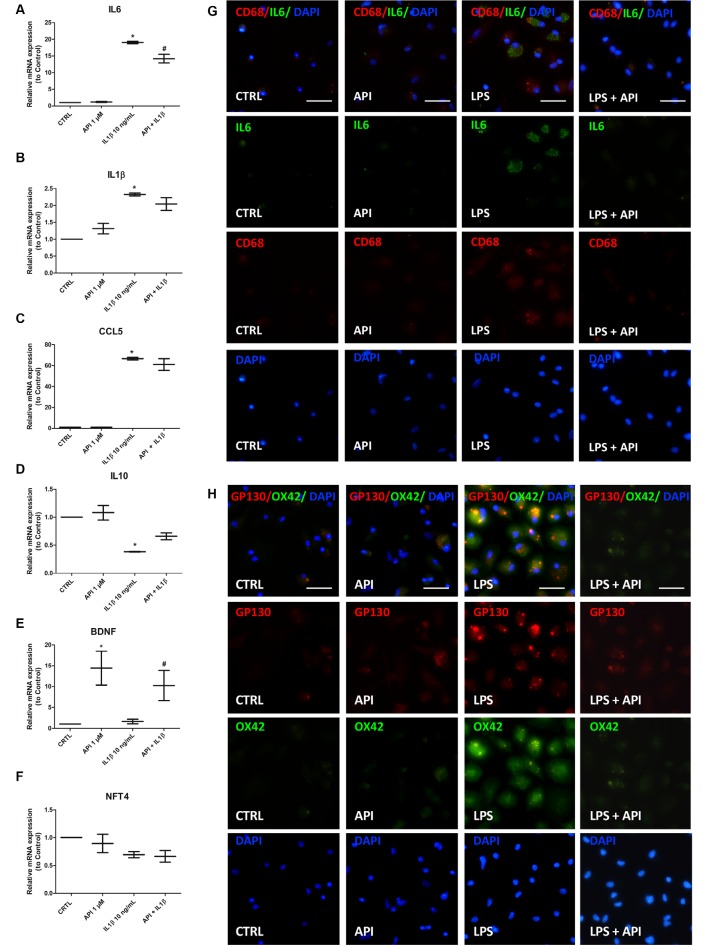
Apigenin regulates the expression of inflammatory mediators. Effects of apigenin (API, 1 μM) treatment on expression of inflammatory and neurotrophic factors by qRT-PCR in glia/neurons co-cultures treated with IL-1β (10 ng/ml). **(A–F)** mRNA expression for proinflammatory (IL-6, IL-1β) and regulatory interleukins (IL-10), for chemokine CCL5, for the brain-derived neurotrophic factor (BDNF) and neurotrophin-4 (NTF-4) were evaluated 24 h after treatment with apigenin. Effects of apigenin (API, 1 μM) treatment on expression of inflammatory markers by immunocytochemistry in glia/neurons co-cultures treated with LPS (1 μg/ml). **(G)** Immunocytochemistry for IL-6 (green), associated with CD68 (red), marker of activated microglia/macrophages in a proinflammatory profile. **(H)** Immunocytochemistry for OX42 (green), associated with gp130 (red), marker of activated microglia/macrophages in a proinflammatory profile. Data were presented as a median of the relative expression to control cultures *statistical significance in relation to the control (DMSO 0.01%), *p*-value < 0.01; ^#^statistical significance in relation to the group treated with IL-1β, *p*-value < 0.01. Were tested for significance using the Kruskal–Wallis test followed by Dunn’s test.

To better characterize the anti-inflammatory effect of apigenin, co-cultures were exposed to IL-1β (10 ng/ml) for 24 h and then treated with apigenin (1 μM). After treatments, the mRNA expression levels of the interleukin 6 (IL-6), C-C chemokine ligand 5 (CCL5) and IL-1β pro-inflammatory mediators, the IL-10 regulatory cytokine, the BDNF and neurotrophin-4 (NTF4) were evaluated by RT-qPCR (Figure 5). After IL-1β damage induction, there was an increase in IL-6, CCL5, and IL-1β mRNA expression when compared to control (Figures 5A–C). After IL-1β damage followed by apigenin treatment, a reduction in IL-6 levels, when compared to cultures treated with IL-1β alone (Figure 5A). Although apigenin treatment induced a reduction in CCL5 and IL-1β gene expression, it was not significant when compared to cultures treated with IL-1β alone (Figures 5B,C). It was also observed a reduction in IL-10 expression after IL-1β treatment, when compared to the control (Figure 5D), and although apigenin treatment induced an increase in IL10 expression levels, it was not significant when compared to cultures treated with IL-1β alone. Moreover, in co-cultures treated only with apigenin the mRNA expression for both pro-inflammatory and regulatory factors was not changed. Furthermore, it was observed that apigenin was able to increase BDNF mRNA expression (14.4) when compared to control with DMSO (0.01%; 1.0; Figure 5E). Our results revealed that apigenin was able to induce increase BDNF mRNA levels in neuronal and glial cell co-cultures after damage with IL-1β (10.22) when compared to the IL-1β-treated group and the control condition (Figure 5E). Neurotrophin NTF4 levels were also evaluated but did not show alterations between groups (Figure 5F). Since IL-1β induces microglial activation and increases IL-6 mRNA expression, we investigated the direct effect of apigenin on inflammatory markers expression in neurons/glial cells co-cultures submitted to LPS stimulus. Immunofluorescence for CD68 (microglial M1 pro-inflammatory marker) and IL-6 (pro-inflammatory cytokine, M1 marker), OX42 (microglial activation marker) and Glycoprotein 130 (gp130), a type 1 cytokine receptor that is within the IL-6 receptor family), showed that inflammatory stimuli increased OX42, IL-6 and gp130 positive cells, which was abolished by apigenin treatment (Figures 5G,H).

### Apigenin Protects Neurons and Reduces Astrocyte and Microglial Activation Induced by Amyloid-β

**Figure 6:**
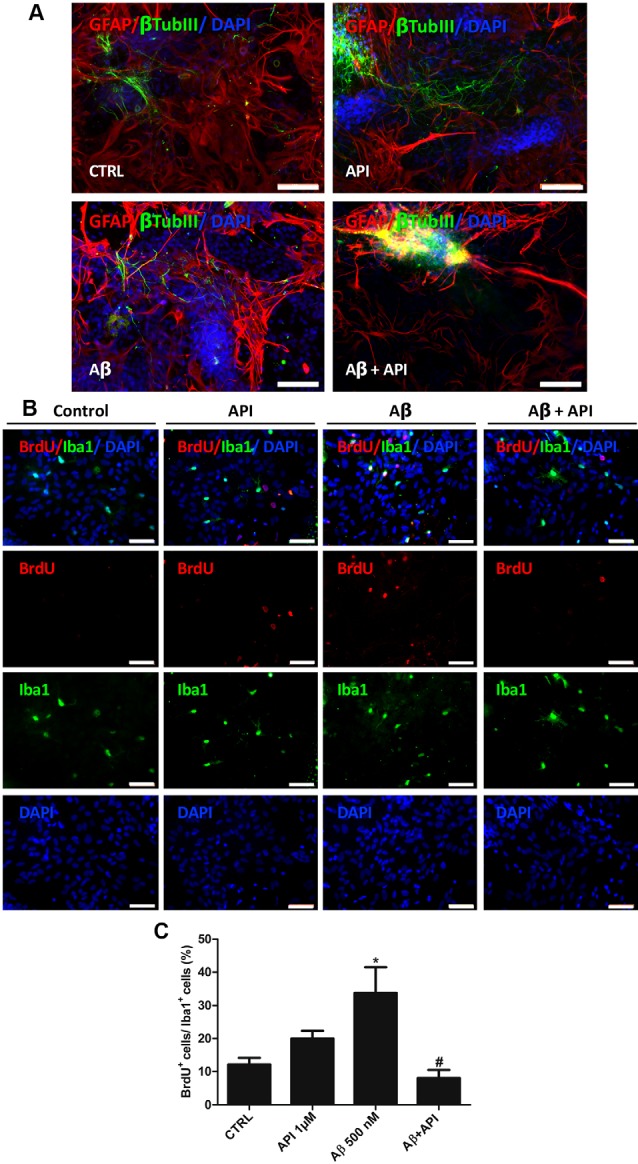
Apigenin protects neurons and reduces glial activation induced by amyloid-β. Effects of apigenin (API, 1 μM) treatment on the integrity of neurons and glial reactivity after Aβ (500 nM) exposure. **(A)** The patterns of expression of the cytoskeletal protein β-tubulin III (β-tubIII, green) specific of neurons, and the cytoskeletal protein GFAP (red) marker of astrocyte morphology and reactivity were determined by immunocytochemistry analysis after 24 h treatments; control cultures were treated with DMSO (0.01%) and nuclear chromatin was stained with DAPI (blue). Obj. 20×, scale bar: 100 μm. **(B)** Immunocytochemistry for ionized calcium-binding adapter molecule 1 (Iba-1, green), specific of microglia, associated with BrdU (red), that marker cells in proliferation was performed in cultures submitted to Aβ. Obj. 40×, scale bar: 50 μm. **(C)** Graph represents cell population labeled for both Iba-1 and BrdU; data are presented as mean of percentage ± standard deviation of immunofluorescence labeling of Iba-1 and BrdU cells. *represents statistical significance in relation to the control (DMSO 0.01%) with *p*-value < 0.05; ^#^represents statistically significant difference compared to the group treated with Aβ, with *p*-value < 0.01. Values are expressed as the mean ± SEM (*n* = 3) and were tested for significance by ANOVA followed by the Newman–Keuls test.

Neuroinflammation in the brain is a feature in AD, this chronic inflammation is mainly attributed to microglial activation and the release of numerous inflammatory mediators (Kinney et al., 2018). We investigated if apigenin can protect neurons against Aβ toxicity and if it reduces microglia and astrocyte activation. After induction of damage with Aβ (500 nM), the labeling for βtubIII was scarce and the astrocytes showed prolongations with high GFAP immunoreactivity, also suggesting glial reactivity (Figure 6A). However, apigenin treatment after Aβ induced damage was able to increase the intensity of βtubIII labeling with the formation of neural clusters and preserved astrocyte morphology and GFAP expression, patterns similar to control cultures (Figure 6A). Apigenin treatment alone did not affect βtubIII expression in neurons and on GFAP expression and astrocyte morphology. Evaluation of microglial proliferation in co-cultures of neurons and glial cells exposed to Aβ oligomers (500 nM) for 4 h and analyzed after additional 24 h demonstrated that Aβ damage promoted microglial proliferation, with an increase in the percentage of Iba-1 and BrdU positive cells (33.7 ± 7.7%) compared to the control (12 ± 2%; Figures 6B,C). However, apigenin treatment after Aβ damage significantly reduced the percentage of positive Iba-1 and BrdU cells when compared to co-cultures treated with Aβ alone and did not show statistical significance in the percentage of these cells when compared to control. Moreover, apigenin treatment (1 μM) had no significant effect on the proportion of double-labeled cells for Iba-1 and BrdU compared to control co-cultures.

## Discussion

Many *in vitro* models of CNS studies have been developed to elucidate the mechanisms associated with insults that lead to neuron death, and thus find better therapeutic targets against neurodegenerative diseases associated with neuroinflammation. Among these models, we highlight the co-culture of neuron and glia, which has several advantages, especially because it is a method that uses high cell density, which may favor the generation of neuronal phenotypes that actively interact with glial cells and mimic tissue-like biological conditions (Al-Ali et al., 2017).

In this study, we used as experimental model co-cultures of neurons and glial cells already well established in our group (Silva et al., 2013) to investigate the anti-inflammatory and neuroprotective potential of flavonoid apigenin in three different inflammatory models: induced by LPS (classical neuroinflammation), IL-1β or Aβ oligomers. In this work, we demonstrate that apigenin is not neurotoxic at the concentration tested and has neuroprotective potential, evidenced by the decreased number of caspase-3^+^ cells and increased neuronal viability. In a previous study, we tested the apigenin (10 μM) activity on embryonic and human-induced pluripotent stem cells (hPSC). Our data showed that apigenin induced neural differentiation and promote synaptogenesis (Souza et al., 2015). Furthermore, in an animal model of spinal cord injury, it was observed that apigenin treatment (10 and 20 mg/kg; *via* ip) recovered neuronal function, and neuroprotective effect associated with antiapoptotic effects, with reduced caspase 3 expression and an increased ratio of Bcl-2/Bax genes (Zhang F. et al., 2014).

In AD, evidences suggest that persistent activation of microglia and astrocytes, initially triggered by Aβ oligomers, triggers a chronic inflammatory response that is partly characterized by the exacerbated production of proinflammatory cytokines. In turn, these cytokines perpetuated neuroinflammation and glial activation and consequently contributed to the progression of neurodegeneration (Stewart et al., 2010; Heneka et al., 2015). These data support the hypothesis that the use of anti-inflammatory agents may slow the progression of this pathology (Daniels et al., 2016). In this sense, apigenin deserves prominence within the group of plant-derived polyphenolic compounds, as it demonstrates anti-inflammatory and neuroprotective potential, presenting itself as a promising compound for the treatment of neurodegenerative diseases, such as AD (Balez et al., 2016; Anusha et al., 2017).

Different CNS stimuli may induce activation, proliferation, and changes in the morphology and function of microglia, which can be modulated by anti-inflammatory agents, such as flavone luteolin, which has been shown to promote change in LPS-exposed BV2 microglial cell lines, from amebic morphology to branched morphology. This change in morphology has also been associated with inhibition of NO synthesis and IL-6 mRNA expression, favoring the M2 phenotype with anti-inflammatory and neuroprotective characteristics (Dirscherl et al., 2010). The findings found in this article were similar to this study, considering that the activation state and the microglia phenotype may be reflected by its cellular form (Vinet et al., 2012; Tang and Le, 2016), we show that apigenin it acts as a potent modulator of the microglial profile since, after induced inflammatory response, apigenin treatment generated morphological change from the ameboid state to branched microglia.

Dynamic changes in microglial phenotypes are associated with neurodegenerative diseases, and the dichotomy of the M1/M2 microglial profile is widely accepted, where such microglia can perform proinflammatory (M1) or anti-inflammatory (M2) functions (Tang and Le, 2016). As already described, LPS (Kobayashi et al., 2013) and Aβ oligomers (Maezawa et al., 2011; Shi et al., 2017; Taipa et al., 2017) are potent inducers of microglia activation, characterized by proliferation and polarization of the M1 microglial profile, characterized by expression of NFκB and CD68 markers. In this study, it was observed that apigenin was able to decrease the percentage of CD68 and BrdU positive (proliferating) microglia, as well as reduced the protein levels of IL-6, OX42 and the gp130 (Type 1 cytokine receptor) after inflammatory stimuli.

Like microglial cells, astrocytes respond to CNS injury, play an important role in neuroinflammation, homeostasis impairment, and synaptic dysfunction observed in AD (Verkhratsky et al., 2010). As described by Ledo et al. (2013), intracerebroventricular injection of Aβ oligomers in mice induces an increase in astrocyte reactivity with increased expression of GFAP in the hippocampus and cortex of these animals and increased levels of proinflammatory cytokines. In the present study, the different inflammatory stimuli induced changes in astrocyte morphology. However, after treatment with apigenin, no morphological characteristics of astrogliosis were visualized. The present study reaffirms the immunomodulatory potential of apigenin and the reduction of IL-1β-induced neuroinflammation, observed through the downregulation of mRNA expression and protein levels of IL-6 a proinflammatory cytokine. Associated with the reduction of IL-6 we also observed a reduction in the protein levels of gp130, identified as the β subunit of the IL-6R complex. Thus, gp130-related cytokine plays an integral role in inflammation (Jones et al., 2011). Similarly, Zhang X. et al. (2014) demonstrated that apigenin (6.25, 12.5 and 25 μM) was able to inhibit the production of IL-6, IL-1β, and CCL5 by human (THP-1) and mouse (J774A.1) macrophages activated by LPS (100 ng/ml) by modulating intracellular signaling pathways as mitogen-activated protein kinase (MAPK), suppressing ERK1/2 phosphorylation and blocking NF-κB activation. The same authors revealed that apigenin suppressed IL-1β production by blocking the activation of caspase-1 and disrupting assembly of the NLRP3 inflammasome complex. Also, they observed that the downregulation of IL-10 by LPS was reversed by apigenin, suggesting that flavonoids may modulate the inflammatory response through multiple mechanisms.

According to Tong et al. (2008), IL-1β interferes with BDNF signaling by suppressing the activation of signal transduction pathways (PI3-K/Akt and MAPK/ERK) that is associated with neuronal survival. Thus, IL-1β makes neurons vulnerable to degeneration by interfering with BDNF-induced neuroprotection. This neurotrophin also plays a critical role in the pathophysiology of AD, in which neuroinflammation is observed, and partly characterized by increased pro-inflammatory cytokines such as IL-1β. Zhang C. et al. (2014) showed that the deregulation of BDNF signaling pathways correlates with synaptic loss and cellular dysfunction underlying cognitive impairment in AD. Our results revealed that apigenin was able to induce increase BDNF mRNA levels in neuronal and glial cell co-cultures after damage with IL-1β. These findings reaffirm the neuroprotective effect of apigenin, as described by Zhao et al. (2013) which demonstrated increased BDNF levels and the restoration of learning deficits and cognitive function in apigenin-treated 2xTg-AD rats.

Taken together, our data suggest that apigenin at a concentration of 1 μM has neuroprotective and anti-inflammatory potential demonstrated in different inflammatory models. Exacerbated inflammatory response to different stimuli was observed, which was characterized by the expression of high levels of proinflammatory cytokines and chemokines, microglial proliferation, polarization to M1 microglial profile and astrogliosis. Therefore, we suggest that this neuroinflammation led to neurodegeneration, which was attenuated by increased BDNF levels and modulation of the inflammatory response as a consequence of apigenin treatment. Finally, this is the first report of the interaction of flavonoid apigenin in the co-culture of neurons and glial cells subjected to IL-1β damage, correlating with AD. In the studies developed by Choi et al. (2014), no neuroprotective effect of apigenin was observed at 30 μM concentration in *in vitro* model of AD using Aβ (25–35) at a concentration of 20 μM. However, in this work, we demonstrated that apigenin in a lower concentration had a neuroprotective and anti-inflammatory effect in different neuroinflammation models.

Evidence indicates that IL-1β expression is one of the most important neuropathological factors in ND, such as AD, being recognized as a central factor in neuroinflammation (Azizi and Mirshafiey, 2012; Xie et al., 2015). Second (Halle et al., 2008) the activation of the NLRP3 inflammasome is closely associated with caspase 1 activation and IL-1β release by Aβ-exposed microglia. In a more recent study (Dempsey et al., 2017) analyzed the effect of small NLRP3 inhibitor molecules called MCC950 on microglia cultures. MCC950 inhibited caspase-1 activation, stimulated Aβ phagocytosis and reduced IL-1β expression *in vitro*. The same study demonstrated, an *in vivo* model using 2xTg-AD mice, that MCC950 (10 mg/kg) also inhibited NLRP3 and microglial activation as well as reduced Aβ accumulation. Together, these data suggest that Aβ accumulation may be mediated by the formation of the inflammasome complex and induction of IL-1β.

Thus, the search for new drugs aimed at blocking the exacerbated formation of this complex and attenuating neuroinflammation may prove to be a valuable strategy in the treatment of ND, including AD. Thus, this work is a pioneer in demonstrating the influence of apigenin on the protection against deleterious effects induced by IL-1β in co-cultures of neurons and glial cells.

## Conclusion

We have shown that apigenin presents neuroprotective and neuroimmunomodulatory effects in *in vitro* models of neuroinflammation. Thus, might represent a potential agent for the treatment of neurodegenerative conditions.

## Data Availability Statement

All datasets generated for this study are included in the article.

## Ethics Statement

The animal study was reviewed and approved by Ethical Committee for Animal Experimentation of the Health Sciences Institute (CEUA, Protocol n°027/2012).

## Author Contributions

ND performed all experimentation, analyzed, interpreted the data and was a major contributor in writing the manuscript. CS, MA, and ABS helped with the maintenance of the cell culture and, additionally, ABS, MA, and BS helped to perform and analyze RT-qPCR. ADA, JS, and DS designed and performed experiments to obtain Aβ oligomers. MC, VS, BS, ADA, DS, and CS revised it critically for intellectual content. CS, AB, and SC designed the experiments, supervised the study, edited and reviewed the manuscript. All authors read and approved the final manuscript.

## Conflict of Interest

AB is a share-holder in the company “GliaGenesis Limited”. The remaining authors declare that the research was conducted in the absence of any commercial or financial relationships that could be construed as a potential conflict of interest.
